# Inferring Whole-Organism Metabolic Rate From Red Blood Cells in Birds

**DOI:** 10.3389/fphys.2021.691633

**Published:** 2021-07-16

**Authors:** Kasja Malkoc, Stefania Casagrande, Michaela Hau

**Affiliations:** ^1^Research Group for Evolutionary Physiology, Max Planck Institute for Ornithology, Seewiesen, Germany; ^2^Department of Biology, University of Konstanz, Konstanz, Germany

**Keywords:** avian erythrocytes, glucocorticoids, aerobic metabolism, respirometry, stress response, mitochondria

## Abstract

Metabolic rate is a key ecological variable that quantifies the energy expenditure needed to fuel almost all biological processes in an organism. Metabolic rates are typically measured at the whole-organism level (woMR) with protocols that can elicit stress responses due to handling and confinement, potentially biasing resulting data. Improved, non-stressful methodology would be especially valuable for measures of field metabolic rate, which quantifies the energy expenditure of free-living individuals. Recently, techniques to measure cellular metabolic rate (cMR) in mitochondria of blood cells have become available, suggesting that blood-based cMR can be a proxy of organismal aerobic performance. Aerobic metabolism actually takes place in the mitochondria. Quantifying cMR from blood samples offers several advantages such as direct estimates of metabolism and minimized disturbance of individuals. To our knowledge, the hypothesis that blood-based cMR correlates with woMR has not yet been directly tested. We measured cMR in red blood cells of captive great tits (*Parus major*), first during their morning activity period and second after subjecting them to a 2.5 h day-time respirometry protocol to quantify woMR. We predicted cMR to decrease as individuals transitioned from an active to a resting state. In the two blood samples we also assessed circulating corticosterone concentrations to determine the perceived disturbance of individuals. From respirometry traces we extracted initial and final woMR measures to test for a predicted positive correlation with cMR measures, while accounting for corticosterone concentrations. Indeed, cMR declined from the first to the second measurement. Furthermore, woMR and cMR were positively related in individuals that had relatively low corticosterone concentrations and displayed little locomotor activity throughout respirometry. By contrast, woMR and cMR covaried negatively in birds that increased corticosterone concentrations and activity levels substantially. Our results show that red blood cell cMR represents a proxy for woMR when birds do not display signs of stress, i.e., either before increases in hormonal or behavioral parameters have occurred or after they have abated. This method represents a valuable tool for obtaining metabolic data repeatedly and in free-living individuals. Our findings also highlight the importance of accounting for individual stress responses when measuring metabolic rate at any level.

## Introduction

Aerobic metabolism is a cornerstone of physiological research because of its fundamental function in fueling most biological processes in aerobic organisms. The importance of metabolic rate (the rate at which energy is produced while consuming oxygen and substrates) for ecological and evolutionary concepts is nowadays universally acknowledged (Glazier, 2015; Gangloff et al., 2020). Indeed, variation in metabolic rate has been linked to life-history traits like growth, reproductive output, and survival (Pettersen et al., 2019; Boyce et al., 2020), as well as behavioral traits (Biro and Stamps, 2010; Holtmann et al., 2017; Mathot et al., 2019) and hormonal profiles (Haase et al., 2016). Metabolic rate is also thought to be a key mediator of the fast-slow life-history continuum (Jimenez et al., 2014a; Auer et al., 2018; Chung et al., 2018) and to underlie various life-history tradeoffs (e.g., those between oxidative damage and longevity: Speakman et al., 2015; or sleep and predation: Ferretti et al., 2019).

Metabolic rate is typically measured as the amount of oxygen (O_2_) that an aerobic organism consumes per unit of time, thus representing an indirect measure of energy expenditure. Oxygen consumption is typically measured in intact organisms through indirect calorimetry, a technique better known as respirometry (Lighton, 2008). Respirometry protocols usually require an animal’s confinement in a sealed (and usually small) metabolic chamber (McNab, 2006), where the composition of both the incoming and outgoing air is recorded continuously to determine an individual’s whole-organism metabolic rate (hereafter woMR). A prolonged confinement of several hours represents a major limitation of respirometry because it may be stressful for the individuals and may lead to biased results (Careau et al., 2008; Murray et al., 2017; but see e.g., Breuner et al., 1999; Wikelski et al., 1999). Also, removing an individual from the wild for respirometry can be prohibitive during certain life history stages like critical reproductive phases and for vulnerable species. Furthermore, to address ecologically relevant questions we need to quantify metabolic rates in free-living individuals as they go about their natural daily activities, i.e., measure “field” or “active” metabolic rates (Butler et al., 2004; Mitchell et al., 2015; Hicks et al., 2020). However, available field methods like the doubly-labeled water technique are often impractical (requiring repeated captures of individuals on subsequent days) or error-prone (Butler et al., 2004; Speakman and Hambly, 2016), emphasizing that we urgently need a more direct and less intrusive methodology.

One method that may circumvent these issues is to measure metabolic rate at the cellular level (“cellular metabolic rate,” hereafter cMR; Koch et al., 2021), which quantifies the respiratory processes directly occurring in the mitochondria, the cell organelles where oxidative phosphorylation takes place. The idea that cMR may deliver information about whole-organism performance comes from biomedical research (Gnaiger, 2009), where the measurement of blood cMR has become increasingly common because samples can be obtained less invasively and, unlike other tissues, blood cells can be used without being altered (i.e., they do not need to be permeabilized to measure respiration rates; Stier et al., 2017). For example, cMR measured in human blood cells like platelets has been shown to reflect variation in organismal-level traits like age, locomotor performance or exercise habits (Tyrrell et al., 2015; Liepinsh et al., 2020). Non-mammalian vertebrates like birds are particularly valuable study systems because they possess nucleated red blood cells (RBCs) that contain functional mitochondria (Stier et al., 2013), allowing the quantification of cMR from a small blood sample. It has been shown that RBC metabolic rate can change with life-history stages (Stier et al., 2019a) and following exposure to stressors (Casagrande et al., 2020).

Studies on birds and fish have provided some support for an association between woMR and cMR, although cMR was measured indirectly via mitochondrial enzymatic activity in several tissues (Salin et al., 2016; Gutiérrez et al., 2019). cMR also varies as a function of different life histories in a similar way as woMR (Jimenez et al., 2014b; Koch et al., 2021), and blood-based cMR is associated with skeletal-, muscle-, and brain-based cMR (Tyrrell et al., 2016, 2017; Stier et al., 2017). Taken together, these findings suggest that cMR measured in a systemic tissue like blood correlates with cMR in other tissues and may be informative of metabolic rate at the whole-organism level. Nevertheless, to the best of our knowledge, a direct relationship between woMR and cMR measured in RBCs has never been established.

Using captive great tits (*Parus major*) as model, we experimentally tested the hypothesis that cMR obtained from red blood cells can serve as a proxy for the metabolic state of an organism using a repeated-measures design. We first predicted that individuals have a higher cMR when they are active and in an absorptive state (i.e., when birds move freely within the aviary and have *ad libitum* access to food) than when resting and being in a post-absorptive state (i.e., after being recorded in a metabolic chamber for 2.5 h without access to food). The recording of cMR and woMR in close temporal proximity in the same individual allowed us to test our second prediction of a positive relationship between cMR and woMR. By recognizing that the extended confinement required for respirometry can be stressful for individuals (Careau et al., 2008; Murray et al., 2017), we also monitored circulating concentrations of corticosterone, which increase in response to unpredictable disturbances (Romero and Wingfield, 2015). Corticosterone concentrations and metabolic rate show parallel increases in response to perturbations in external and internal conditions in birds, both at the organismal (Jimeno et al., 2017) and the cellular level (Casagrande et al., 2020). Therefore, we hypothesized that individual stress responses (circulating corticosterone concentrations as well as a behavioral expression of stress-locomotor activity displayed during respirometry) might play a role in the relationship between woMR and cMR. Specifically, since we expected hormonal and behavioral stress responses to vary among individuals (Baugh et al., 2013), we predicted that individuals with more pronounced elevations between the two sampling points (i.e., displaying strong increases in corticosterone concentrations and activity levels during the experimental procedure) would also show increases in woMR and cMR over time.

## Materials and Methods

### Experimental Design

The experiment was conducted in December 2019 over the course of 8 days on 21 hand-raised great tits (11 males and 10 females) housed singly in outdoor aviaries (L×W×H: 400 × 100 × 220 cm). Birds were provided with food (seeds, mealworms, wax moths, suet balls, and a minced beef heart mixture) and water *ad libitum*.

**FIGURE 1:**
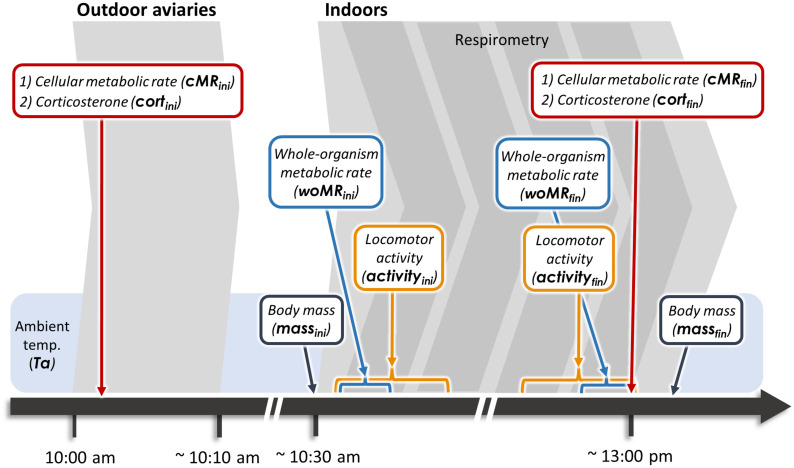
Experimental time line. Red boxes indicate measures obtained from blood samples, while blue boxes refer to woMR measurements. Blue brackets indicate 10-min periods over which woMR was averaged, while orange brackets indicate the 1-h periods over which locomotor activity was quantified. Ta, ambient temperature.

Each morning between 10:00 and 13:00 (to allow the birds to feed prior to the start of the experiment) three individuals were caught and subjected to the measurement protocol. Each individual was blood sampled twice, i.e., before and after a 2.5-h respirometry session (Figure 1).

Within 3 min (mean ± SD: 146 ± 63 s) from entering the aviaries, we collected an 80 μL blood sample from the wing vein to determine baseline corticosterone concentrations and cMR. Ambient temperature inside the aviaries was recorded (mean ± SD: 1.4 ± 2.5°C) before bringing birds indoors. Each bird was weighed and then placed into a 1 L perch-equipped chamber located inside an environmental cabinet. The temperature inside the environmental cabinet was set to equal that recorded in the outdoor aviaries at capture to minimize effects of thermal acclimation on metabolic and hormone measurements. The birds’ behavior was recorded with infrared mini-cameras (Handykam, Sony HD, HK100441w, Redruth, United Kingdom) during the entire respirometry session to quantify locomotor activity (“activity,” see Supplementary Text 3 for details on quantification and analysis of activity). At the end of each session, we collected a second blood sample within 3 min (mean ± SD: 175 ± 19 s) from entering the experimental room, before weighing the individual again and releasing it back into the outdoor aviary. All experimental procedures were conducted according to the legal requirements of Germany and were approved by the governmental authorities of Oberbayern, Germany.

### Metabolic Measures

Prior to the current experiment, we determined the lower critical temperature for our study population to be ∼15°C (Supplementary Figure 1). Hence, cMR and woMR were both recorded well below thermoneutrality. However, some metabolic rate measurements were taken when individuals were in different activity and digestive states. Initial cMR (from blood samples collected in the aviaries) was obtained from individuals in a naturally active state, thus being more similar to field metabolic rate, which quantifies the energy metabolized by free-ranging individuals (Butler et al., 2004). By contrast, all metabolic rate measurements collected indoors (both woMR and final cMR) were taken from individuals in a state of forced resting and are more similar to those of standard metabolic rate (which is measured at rest but also includes thermoregulatory costs; Brett and Groves, 1979). Furthermore, initial and final measurements also differ with respect to digestion: for the initial metabolic rate measurements individuals were in an absorptive state (after having fed in the aviaries), while the final measurements reflect a post-absorptive state since neither food nor water were provided in the metabolic chambers. For reasons of simplicity, we refer to our metabolic measures with respect to the scale at which they were taken, i.e., as cellular and whole-organism metabolic rate (cMR and woMR, respectively), adding the subscripts “**ini**” or “**fin**” to specify whether they represented initial or final measures for each individual (Figure 1). Importantly, final measures for cMR, woMR, and corticosterone were collected in close temporal proximity (within ∼10 min), while this was logistically not possible for all initial measures. In fact, cMR_ini_ and cort_ini_ were collected simultaneously, but woMR_ini_ was quantified about 40 min later (mean ± SD: 39.0 ± 7.0 min), which is the time required to blood sample the birds, bring them indoors, weigh them, place them in metabolic chambers and initiate the recordings.

### Quantification of Hormone Concentrations

Blood samples were immediately centrifuged and plasma stored at −20°C until analysis (in January 2020). Corticosterone concentrations were measured in duplicate using an enzyme immunoassay (Arbor Assays, Catalog No. K014-H5) following a double diethyl ether extraction (Casagrande et al., 2020). Samples and quality control samples (Supplementary Text 1) were measured in duplicates, with samples from the same individual being assayed in adjacent wells on the same assay plate, while samples from different individuals were randomly distributed over two plates. Inter-assay variance for the low quality control was 14%, and for the high quality control was 3%. Intra-assay variance for the low quality control was 6%, and for the high quality control 4%.

### Quantification of Whole-Organism Metabolic Rate

Whole-organism metabolic rate was measured with flow-through respirometry using three independent, but identical units (Sable Systems, Las Vegas, NV, United States). Each unit comprised a metabolic chamber consisting of a 1 L stainless steel cylinder with an air-tight lid, situated inside a temperature-controlled environmental cabinet (Binder, KB53 E3.1, Tuttlingen, Germany) where ambient temperature was adjusted daily to the ambient temperature recorded earlier in the outdoor aviaries (see section “Experimental Design”). In each respirometry unit, water vapor, CO_2_, and O_2_ were analyzed downstream of both the chamber and the baseline circuits. H_2_O and CO_2_ were removed from incurrent air using Drierite (Sigma-Aldrich, Darmstadt, Germany) and Ascarite (Thermo Fisher Scientific, Schwerte, Germany) scrubbing columns and syringes, while a constant 500 mL min^–1^ air flow was maintained by mass-flow controllers (MFS, Sable Systems, Las Vegas, NV, United States). Excurrent air was subsampled and the concentrations of water vapor (RH-300 WVA, Sable Systems, Las Vegas, NV, United States), CO_2_, and O_2_ (Foxbox, Sable Systems, Las Vegas, NV, United States) were analyzed.

The respirometry sampling scheme was created in Expedata (Sable Systems, Las Vegas, NV, United States) prior to data collection. The total duration of the recording was set to 3:00 h, and divided into segments as follows: the first 10 min were recorded from the baseline circuit, the following 2:20 h were recorded from the chamber circuit and during the last 30 min the system switched back to recording the baseline stream. The recording was initiated immediately upon placing the birds into the metabolic chambers. Birds were removed from the metabolic chamber as soon as the 2:20 h of recordings ended. This strategy allowed us to blood sample the birds immediately after the end of the respirometry (see main text and Figure 1), while the respirometry equipment was automatically recording the last baseline period. Prior to initiating each recording, we ventilated the experimental room well to ensure a high O_2_ concentration in the room air (we also kept windows open during the entire measurement). Furthermore, the respirometry equipment was turned on (set to the baseline circuit) each day at least 30 min before bringing the birds indoors. This allowed the air to freely circulate within each component and to obtain a stable signal at the beginning of the recording. Analogically collected data (1 data point per second) were converted into digital (UI-2 and UI-3, Sable Systems, Las Vegas, NV, United States) and shown as real-time gas traces in Expedata. WoMR_ini_ and woMR_fin_ were calculated as the mean O_2_ consumption per minute (Lighton, 2008) during the first and the last 10 min of the respirometry recording, respectively (Figure 1; see also Supplementary Text 2 for O_2_ consumption calculations).

### Quantification of Cellular Metabolic Rate

The quantity of oxygen consumed by cellular aerobic metabolism was measured in intact red blood cells (RBCs), following Stier et al. (2017). This protocol has previously been validated for great tits (Casagrande et al., 2020). Briefly, cMR was measured in 20–40 μL (mean ± SD: 29 ± 7 μL) of fresh RBCs washed in MiR05 buffer. Immediately before the start of the mitochondrial measurements, washed RBCs were resuspended in 1 mL of MiR05 buffer already equilibrated at 40°C in the respirometry chamber of a Clark-electrode high-resolution respirometer (Oxygraph-2k, Oroboros Instruments, Innsbruck, Austria). We measured cMR as the rate at which O_2_ was consumed during basal respiration (“routine” in Stier et al., 2017). CMR was corrected for non-mitochondrial O_2_ consumption by subtracting the O_2_ consumption measured after adding 5 μM of the mitochondria inhibitor antimycin A. Finally, cMR was normalized by the volume of the RBC aliquot used and expressed as pmol O_2_ s^–1^ per volume of RBCs.

### Statistical Analyses

Statistical analyses were conducted in R (3.5.3; R Core Team, 2013) using a Bayesian approach with non-informative priors. Linear mixed-effects models (LMMs) were employed to first, analyze the internal and external factors that may have affected each of the physiological variables recorded in this study (Supplementary Table 1), second, to describe the relationship between corticosterone and woMR and cMR, respectively (Supplementary Table 2) and third, to model woMR in relation to cMR (Table 1). LMMs were implemented using the “lme4” package (Bates et al., 2015) and the “sim” function from the “arm” package (Gelman and Hill, 2007) to draw 5,000 random values from the joint posterior distribution of the model parameters. 95% Bayesian credible intervals (CrI) were extracted around the mean (Gelman and Hill, 2007), while posterior probabilities associated with specific hypotheses were reported whenever needed. We use the adjective “meaningful” to describe an effect for which its 95% CrI do not include zero and/or when its posterior probability is ≥95% (Korner-Nievergelt et al., 2015). Because of their right-skewed distribution, we log_10_ transformed corticosterone data, both when used as a response and as a predictor variable. This transformation helped normalizing the corticosterone distribution and resulted in better model residuals when used as a response variable, while it assured a linear relationship with the response variable when used as a predictor variable. Furthermore, we also standardized all continuous explanatory variables by mean-centering and standardizing their variances.

**TABLE 1:** Results from a linear mixed-effect model to analyze the relationship between whole-organism metabolic rate (woMR) and cellular metabolic rate (cMR).

	Whole-organism metabolic rate

Fixed factors	β (95% CrI)
Intercept	**2.61** (2.26, 2.94)
cMR	−0.01 (−0.10, 0.09)
Log_10_(cort)	**0.22** (0.09, 0.36)
cMR × log_10_(cort)	**−0.13** (−0.23, −0.03)
Mass	−0.03 (−0.16, 0.09)
T_a_	**−0.23** (−0.34, −0.12)
Measurement (fin)	**−0.29** (−0.57, −0.01)

**Random factors**	**σ^2^ (95% CrI)**

Chamber	0.07 (0.00, 0.26)
Individual ID	0.03 (0.01, 0.05)
Residual	0.03 (0.02, 0.05)

*Statistically meaningful effects are highlighted in bold.*

To test whether woMR was related to cMR, we built a full LMM (Table 1) with woMR as the response variable and including several biologically meaningful variables (Korner-Nievergelt et al., 2015). As predictors in this LMM, we included cMR and corticosterone as well as their interaction (“*cMR* × *cort*”), while also adding measurement (“*measurement*,” initial or final), body mass (“*mass*”) and ambient temperature (“*T*_a_”). Measurement was included to account for any differences in experimental conditions between initial and final measures (i.e., in digestive and activity status), while body mass and ambient temperature were included because of their well-known relationship with metabolic rate (Swanson and Olmstead, 1999; Hudson et al., 2013). Individual ID was added as a random factor to account for the non-independence of the repeated measures of individuals (initial and final). Chamber number (“*chamber*,” a dummy variable coding for the three respirometry units) was also added as random factor to account for differences in woMR values among respirometry units. We included corticosterone and its interaction with cMR because corticosterone levels are known to influence metabolism at both cellular and organismal levels, which is also supported by our own data (Supplementary Table 2). Although the degree of activity can influence metabolic rate values, we could not include locomotor activity in our full model as it showed a strong correlation with corticosterone concentrations (*r* = 0.77; *p* = 0.0001, obtained from a repeated measures correlation with the “rmcorr” package; Bakdash and Marusich, 2017). We also tested whether the association between woMR and cMR was different for initial *versus* final measurements by including the three-way interaction term “*cMR* × *cort* × *measurement*.” Since adding this interaction resulted in a worse model fit and did not produce a meaningful result, we excluded it from the final model. For further details on the potential influence of other variables and on model structuring and diagnostics see Supplementary Text 4.

## Results

### Trait Changes From Initial to Final Measures

**FIGURE 2:**
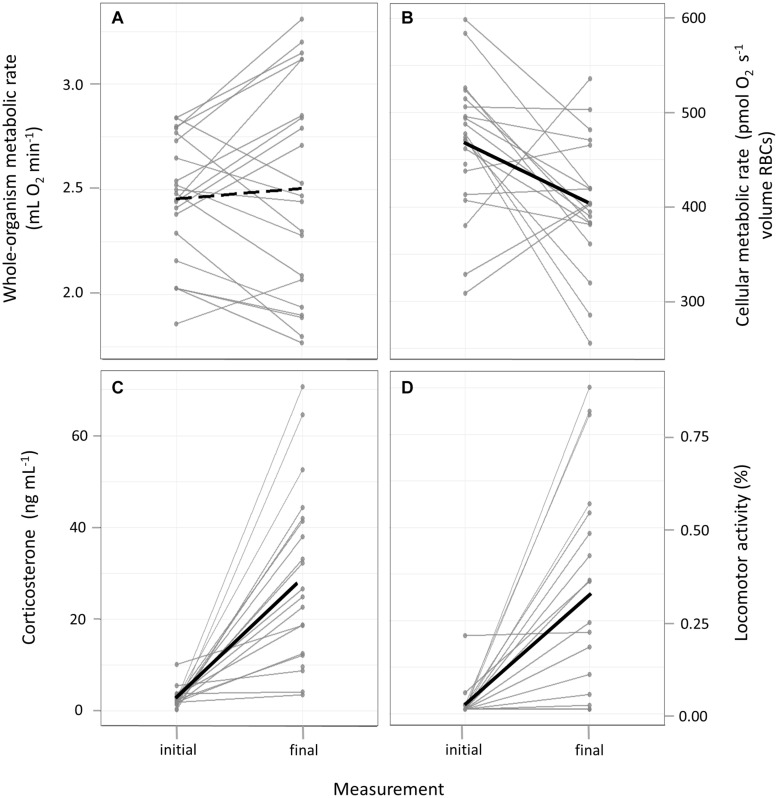
Initial and final measurements of individuals in: **(A)** whole-organism metabolic rate; **(B)** cellular metabolic rate in red blood cells; **(C)** plasma corticosterone concentrations; **(D)** proportion of time displaying locomotor activity. Thin gray lines connect repeated measures of individuals. Thick black lines indicate the mean change in each trait, with solid lines referring to statistically meaningful effects.

On average, individuals increased cort concentrations [β = *0.92*, *CrI* = (*0.68, 1.17*); Supplementary Table 1A and Figure 2C] and the time spent active [β = *1.34*, *CrI* = (*0.99, 2.22*); Supplementary Table 1D and Figure 2D], whilst decreasing cMR [β = *−48.55, CrI* = *(−95.47, −3.00*); Supplementary Table 1C and Figure 2B]. WoMR did not show any meaningful change from initial to final measure (Supplementary Table 1B and Figure 2A), despite varying greatly among individuals. Final corticosterone concentrations and activity levels were positively associated [β = *1.80*; *CrI* = (0.83, *3.15*); Supplementary Table 3A and Supplementary Figure 2A].

### Corticosterone Changes the Association Between woMR and cMR

The positive effect for the interaction “*cort × measurement*” in the LMM (Supplementary Table 2A) indicated that birds that had higher corticosterone concentrations at the end of the respirometry also had higher woMR [β = *0.50*, *CrI* = (*0.22, 0.79*)]. By contrast, there was no association between corticosterone concentrations and cMR at any point during the experiment (Supplementary Table 2B). Corticosterone concentrations were expected to be associated with both woMR and cMR but were instead only related to woMR, further justifying their inclusion in our main model to analyze the relationship between woMR and cMR.

Our full LMM showed indeed a meaningful, but negative, interaction between *“cMR* × *cort”* [β = *−0.13*, *CrI* = (*−0.23, −0.03*), posterior probability = 0.99, Table 1], suggesting that circulating corticosterone concentrations were linked to the relationship between woMR and cMR. This result indicates that when birds had high corticosterone concentrations (i.e., increased corticosterone to a great extent from the initial to the final measurement), their cellular metabolic rate was negatively related to their whole-organism metabolic rate.

**FIGURE 3:**
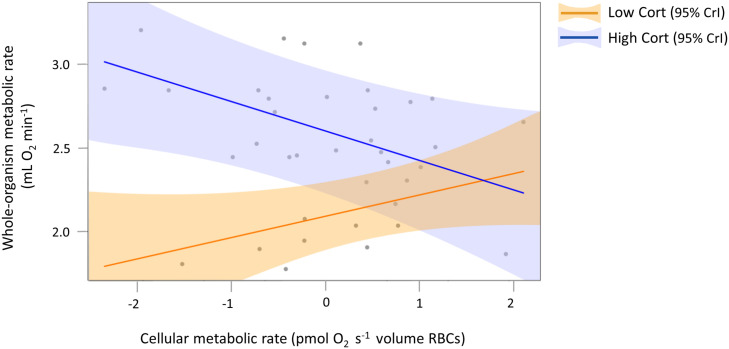
Interaction plot to visualize the opposing relationships between woMR and cMR for two exemplary extreme corticosterone phenotypes. Note that our main result of a significant interaction between the two continuous predictors cMR and cort explaining variation in woMR (Table 1) is based on all data. Here, only two fitted lines, obtained from simulated posterior distributions (see section “Materials and Methods”), are presented to illustrate the relationships between woMR and cMR for individuals with very low (orange) and very high (blue) corticosterone concentrations, at an average ambient temperature and body mass, after accounting for the random effects of ID and metabolic chamber. Gray circles represent raw data (21 individuals measured twice, resulting in a total of 37 data points for which all the physiological parameters were successfully measured), plotted exclusively to show that fitted lines lie within the range of the actual data and that the latter do not cluster tightly around fitted lines, indicating that overfitting has not occurred. Shaded areas around each fitted line represent 95% CrI. Continuous predictors were mean-centered and their variances standardized but for clarity original units were retained in the axis title.

To visualize and further analyze our main finding that variation in woMR was explained by a significant interaction between cMR and corticosterone (Table 1), we focused on modeling two exemplary extreme corticosterone phenotypes in our study population. For this, we used the “sim” function from the R “arm” package (Gelman and Hill, 2007) to simulate Bayesian posterior distributions of the parameter estimates describing our full model (Table 1). We set the number of simulations to 5,000, so that 5,000 independent draws could be obtained for each model parameter. Then we extracted the (simulated) posterior distributions referring to each parameter and we reconstructed the regression equation of our full model, this time by multiplying the distribution describing the interaction term either with the 10% or the 90% quantiles of our actual corticosterone distribution (which correspond to 1.7 and 42.2 ng mL^–1^, respectively). This step resulted in two (simulated) joint posterior distributions of parameter estimates, which described the regression line for the low corticosterone phenotype (the orange line in Figure 3) and that for the high corticosterone phenotype (the blue line in Figure 3), respectively.

Although there are no formal *post hoc* tests in Bayesian analyses, by adopting such a simulation-based approach we were able to calculate the mean, the CrI and the posterior probability associated with the low and high corticosterone effects separately. We found that for low corticosterone concentrations (i.e., when the individual had low or only slightly increased corticosterone concentrations from initial to final measurement) the relationship between woMR and cMR was positive and statistically meaningful [β = *0.13*; *CrI* = (*0.02, 0.28*), posterior probability = 0.95], while for high corticosterone concentrations (i.e., when the individual increased corticosterone from initial to final measure to a large extent) the woMR-cMR association was negative and also statistically meaningful [β = *−0.17*; *CrI* = (*−0.33, −0.02*), posterior probability = 0.98].

## Discussion

In this study, we tested the hypothesis that cellular metabolic rate measured in bird erythrocytes reflects the whole-organism metabolic state. In line with our first prediction, we found a decrease in cMR from the initial to the final measure (Figure 2B), reflecting the birds’ change from an active and absorptive to a resting and post-absorptive state. Our second prediction was also generally confirmed in that we detected a positive association between woMR and cMR in individuals that showed low final plasma corticosterone concentrations and displayed little activity during respirometry (Figure 3, orange regression line), i.e., in birds that either did not display pronounced hormonal and behavioral responses over the course of respirometry, or more likely, managed to terminate their endocrine stress response and return to low concentrations by the end of the procedure. The finding that the relationship between woMR and cMR was associated with corticosterone responses supported our third prediction, although aspects of the relationships between corticosterone and the two metabolic measurements deviated from our predictions (see below). Our results thus indicate that cMR measured in RBCs can be used as a proxy for woMR whenever individuals have low corticosterone concentrations (Table 1), i.e., either when sampled within maximally 3 min after capture (Romero and Reed, 2005) or after the termination of the corticosterone stress response, when concentrations have returned to low levels.

On average, individuals had higher corticosterone concentrations in the initial compared to the final blood sample (Figure 2C), with most of the birds reaching concentrations equal to or exceeding stress-induced levels of a wild population of great tits sampled at a similar time of year (late fall/winter: ∼20 ng mL^–1^; Baugh et al., 2013). Hence, the majority of our study individuals responded to the experimental procedure with an extended endocrine stress response. However, approximately a third of our birds (6 out of 20) had final corticosterone concentrations that were close to initial, baseline values (∼9 ng mL^–1^; Figure 2C, see also Baugh et al., 2013). In birds, a single stressful event typically leads to high stress-induced corticosterone concentrations within 30–60 min (Wingfield et al., 1982), after which levels usually decrease again due to the activation of negative feedback (Romero and Wingfield, 2015; Taff et al., 2018). Thus, individuals with low final corticosterone concentrations likely underwent a full endocrine stress response but activated negative feedback in the middle of respirometry and/or they might have perceived the experimental procedure as a stressor of lower magnitude in comparison to their conspecifics with higher final corticosterone concentrations. This highlights that existing individual differences in hormonal stress responses in natural and captive populations need to be accounted for Ouyang et al. (2011), Cockrem (2013), Guindre-Parker et al. (2019). A closer monitoring of corticosterone concentrations in the current study was not possible for several logistical reasons but would be needed to better understand individual corticosterone patterns during this experiment. Furthermore, it would also be interesting to characterize among-individual differences in HPA responses (concentrations of corticosterone at baseline, at stress-induced levels and following negative feedback) to determine whether these predict physiological and behavioral patterns observed in this study.

Our hypothesis that individual stress responses – if the experimental procedure represented a stressor – were associated with the relationship between woMR and cMR was supported. Great tits with relatively low corticosterone concentrations at the end of respirometry showed the metabolic patterns that we had predicted *a priori*. These “low-cort” individuals displayed low rates of activity during respirometry (Supplementary Figure 2A) and decreased both woMR (Supplementary Figure 4) and cMR (as did most “high-cort” birds, Figure 2B), in line with our expectations. Importantly, in these “low-cort” birds, woMR was positively predicted by cMR (Figure 3, orange line). By contrast, individuals that had final corticosterone concentrations within the stress-induced range showed different patterns. These “high-cort” individuals displayed high rates of activity, particularly during the last hour of respirometry (Supplementary Figure 2A), and increased woMR while decreasing cMR over the course of the experiment, resulting in a negative relationship between these two metabolic traits (Figure 3, blue line). We can offer one fascinating explanation for this result, which involves the effect of catecholamines (i.e., epinephrine and norepinephrine) on cMR. Catecholamines are released within seconds after the onset of a stressor and initiate glycogen breakdown, leading to increased blood glucose concentrations needed to sustain the fight-or-flight response (Nonogaki, 2000; Sapolsky et al., 2000; Romero and Wingfield, 2015; Romero and Beattie, 2021). Besides these immediate actions, epinephrine can also exert prolonged effects on metabolism by accelerating the rate of glucose transport into avian erythrocytes up to three-fold (in domestic geese; Whitfield et al., 1974). Interestingly, this increased glucose transport rate was associated with a marked depletion of cellular ATP content in RBCs within 90 min of epinephrine exposure (Whitfield et al., 1974). Thus, if our “high-cort” individuals also underwent a larger and more extended increase in epinephrine concentrations in response to capture, handling and the respirometry protocol, this could have resulted in a stronger downregulation of RBC cMR as compared to “low-cort” individuals. To test this idea and elucidate the mechanisms that regulate cellular responses to stressors, future studies should measure cMR in avian RBCs together with circulating concentrations of catecholamines and glucocorticoids in individuals exposed to stressors of different duration.

Although we predicted an association between corticosterone and both metabolic rate measures, we only found a positive relationship between final corticosterone and woMR (Supplementary Table 2A and Supplementary Figure 4), i.e., individuals with higher final corticosterone concentrations also had a higher final woMR, confirming patterns reported previously (Haase et al., 2016; Jimeno et al., 2018). We can only speculate as to why we did not observe a positive association between cMR and corticosterone (Supplementary Table 2B). Besides the metabolic dynamics potentially being influenced by catecholamines as discussed above, another reason may be limitations in our data collection. Had we been able to measure corticosterone concentrations and cMR immediately before recording woMR_ini_, we likely would have found stress-induced corticosterone concentrations and perhaps also elevated cMR. Mitochondrial traits are known to be modulated by glucocorticoids during acute stress responses (Manoli et al., 2007; Psarra and Sekeris, 2009). In line with this, in king penguins (*Aptenodytes patagonicus*) 30 min after capture and handling the efficiency of mitochondrial respiration (i.e., ATP production per oxygen and substrate consumed) in RBCs was decreased in response to stress-induced increases in corticosterone (Stier et al., 2019b). That is, even though in this study cMR itself did not change, other aspects of mitochondrial metabolism like proton leak were linked to stress-induced corticosterone concentrations (Stier et al., 2019b). Importantly, this effect was detected only after accounting for individual corticosterone stress responses, like in the present study.

Nonetheless, the fact that we did find a positive association between the final measures of corticosterone and woMR is noteworthy because cort_ini_ and cMR_ini_ were both taken within 3 min from entering the outdoor aviaries (i.e., before stress-induced increases in corticosterone occurred), while our recordings for woMR_ini_ did not begin until ∼40 min later, after birds had been transferred to the respirometry chamber. At that time, corticosterone likely reached stress-induced concentrations (Sapolsky et al., 2000; Baugh et al., 2013) and our woMR_ini_ measures may have already been inflated by this increase in hormone concentrations. This provides a plausible explanation as to why we could not detect an increase in average woMR values from initial to final measures. Instead, the population-level change in woMR resulted in a flat, non-meaningful change (Figure 2A), leading to an apparent incongruence in the corticosterone-woMR association at the individual *versus* population level. We believe that had we been able to measure woMR_ini_ within 3 min from catching, as we were for cort_ini_, the population-level change in woMR would have been an increase, like for corticosterone. Despite this methodological caveat, we detected a positive relationship between corticosterone concentrations and woMR in final measures. This supports the notion that individual differences in corticosterone responses play a major role in the outcome of this study, enabling us to uncover the dependence of the woMR-cMR association on individual stress responses. Our choice of performing a repeated-measures study together with appropriate statistics (LMMs that accounted for individual corticosterone responses) proved decisive for this aim. Indeed, performing separate analyses on initial *versus* final measures would not have yielded meaningful results. As mentioned above, due to the methodological limitations of respirometry, woMR_ini_ reflected a different physiological state than cMR_ini_ and cort_ini_, leading to inaccurate conclusion when examined in isolation. Likewise, analyzing relationships only among final values would not have been informative either because it did not allow to account for individual corticosterone responses.

Obtaining a “resting” (i.e., “non-stressed”) measure of woMR during the active phase of an individual is widely recognized as being challenging. It can be so difficult that “*In most respirometry studies, if an individual never quiets sufficiently to generate a stable* “*resting” MR during a trial, the run is rejected*” (Careau et al., 2008). Researchers usually discard the first part of respirometry measurements because the data are affected by an individual’s acclimation to the metabolic chamber (Careau et al., 2008). In fact, day-time baseline measures of woMR in diurnal species can only be achieved under special circumstances, like when individuals are housed in large metabolic chambers and measuring metabolic rate does not require any handling (Klaassen and Biebach, 1994). Else, metabolic rates are best determined overnight after individuals have calmed down and returned to baseline corticosterone concentrations (Wikelski et al., 1999). But these overnight protocols are performed under somewhat artificial conditions that are rarely encountered by individuals in the wild.

In our study, the average decrease in cMR observed across all individuals from initial to final measurements (Figure 2B), corroborates an important prediction and suggests that cMR in RBCs reflects the metabolic state of an individual. Thus, cMR might be used to obtain real-time snapshots of an individual’s metabolic rate in the field, which is where ecologically relevant processes are taking place. However, we point out that further studies, also using other taxa like reptiles and amphibians, are needed to fully confirm that measuring cMR in RBCs represents a valuable tool to reliably, repeatedly and relatively non-invasively assess metabolic rate in the wild.

In future work, it will also be important to experimentally identify the corticosterone concentrations above which the positive relationship between RBC cMR and woMR turns into a negative one. Note that such a threshold will likely differ among individuals as well as within individuals across contexts. For now, we recommend that users collect cMR samples within the shortest time possible after capture to avoid any bias introduced by increased glucocorticoid concentrations. If cMR cannot be obtained from non-stressed individuals, we recommend the parallel assessment of plasma glucocorticoid concentrations to account for individual endocrine stress responses.

In conclusion, even though evidence of a positive association between woMR and cMR existed (Norin and Malte, 2012; Peña-Villalobos et al., 2014; Salin et al., 2016; Gutiérrez et al., 2019), our study is the first to directly relate oxygen consumption at the cellular level in RBCs to oxygen consumption at the whole-organism level. Our results demonstrate that blood is a versatile systemic tissue that can provide – quickly and repeatedly – metabolic information at the organismal level, with minimal invasiveness. Our findings are appealing not only for studies on free-living individuals, especially during highly sensitive life history stages like reproduction, but also for conducting longitudinal studies using repeated-measures designs. We hope that our results not only raise an awareness that experimental protocols involving traditional respirometry may represent prolonged stressors to individuals, but also stimulate evolutionary biologists, physiologists and ecologists to apply new approaches for studying metabolism and energetics in natural populations.

## Data Availability Statement

The datasets presented in this study can be found in the Dryad Digital Repository (https://doi.org/10.5061/dryad.ncjsxksvh).

## Ethics Statement

The animal study was reviewed and approved by the Governmental authorities of Oberbayern, Germany.

## Author Contributions

KM, SC, and MH conceived and designed the study. KM collected the data, carried out the statistical analysis, respirometry, video analysis, and hormone analysis, and drafted the manuscript. SC participated in collecting data and preliminary statistical analysis, carried out the mitochondria analysis, and revised the manuscript. MH participated in collecting data and revised the manuscript. All authors approved the final version of the manuscript.

## Conflict of Interest

The authors declare that the research was conducted in the absence of any commercial or financial relationships that could be construed as a potential conflict of interest.
