# Supplementary material for: Inferring Whole-Organism Metabolic Rate From Red Blood Cells in Birds
Source: Front Physiol. 2021 Jul 16;12:691633. doi: 10.3389/fphys.2021.691633 (PMC8322697; doi:10.3389/fphys.2021.691633)
Supplement: Supplementary file 1 [file Data_Sheet_1.pdf]

Supplementary Materials

Supplementary Figure 1. Determination of lowest critical temperature (LCT) of the captive population of great tits used in the present study.

Whole-organism metabolic rate was measured overnight in a post-absorptive state in 10 birds (5 males, 5 females) exposed to a gradient of decreasing ambient temperatures (Ta) from 25°C to 5°C, in steps of 5°C (i.e. a total of 5 temperatures). Every bird experienced each temperature for 1.5 h, except for the starting temperature, which was maintained for 2.5 h (to allow the bird to acclimate to the chamber). WoMR was calculated as average O_2 consumption over the last 10 min of each temperature trial. A LMM was run to test whether woMR differed across temperatures, while accounting for repeated measures of individuals, body mass, chamber number, sex and the temporal autocorrelation in woMR across sequential temperature intervals. After finding that temperature had a significant effect on woMR (data not shown), we ran a Tukey-based comparison test to identify the temperature transition following which woMR significantly increased, i.e. when birds began to thermoregulate. This test (data not reported) confirmed that woMR remained unchanged between 25°C and 15°C (i.e. Ta was still within thermoneutrality), while it started to increase significantly when Ta was decreased from 15°C to 10°C and then kept increasing linearly as temperatures decreased further. In the graph, the thick black line represents the average woMR, together with its 95% CI (grey shading) and the vertical blue line indicates the lowest critical temperature (LCT, ~ 15°C), below which any metabolic rate measurement includes thermoregulatory costs in our great tit population.

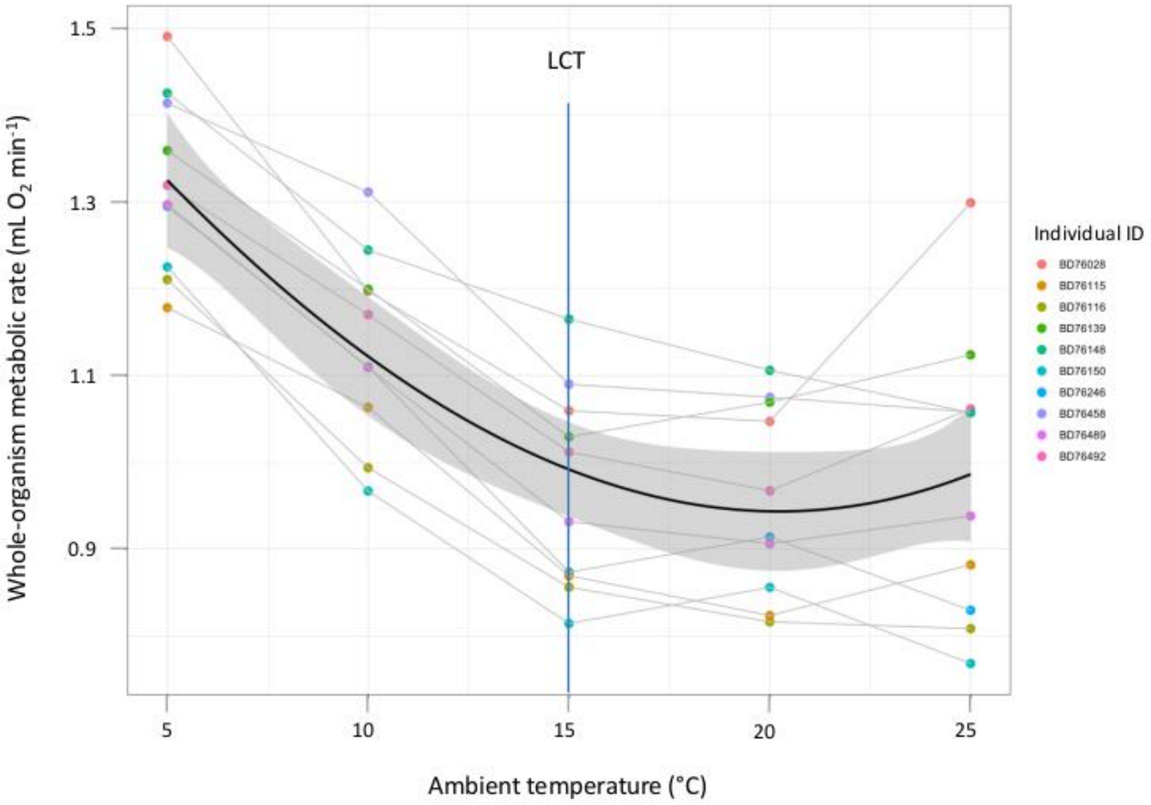

Supplementary Figure 2. Results from GLMMs modelling locomotor activity during respirometry (y-axis) in relation to corticosterone concentrations (**A**), whole-organismal metabolic rate (**B**) and cellular metabolic rate (**C**), respectively. Filled circles and solid regression lines represent initial measures, while filled triangles and dashed lines represent final measures. Asterisks indicate statistically meaningful effects as obtained from the corresponding GLMM using beta regression, as reported in Table 3.

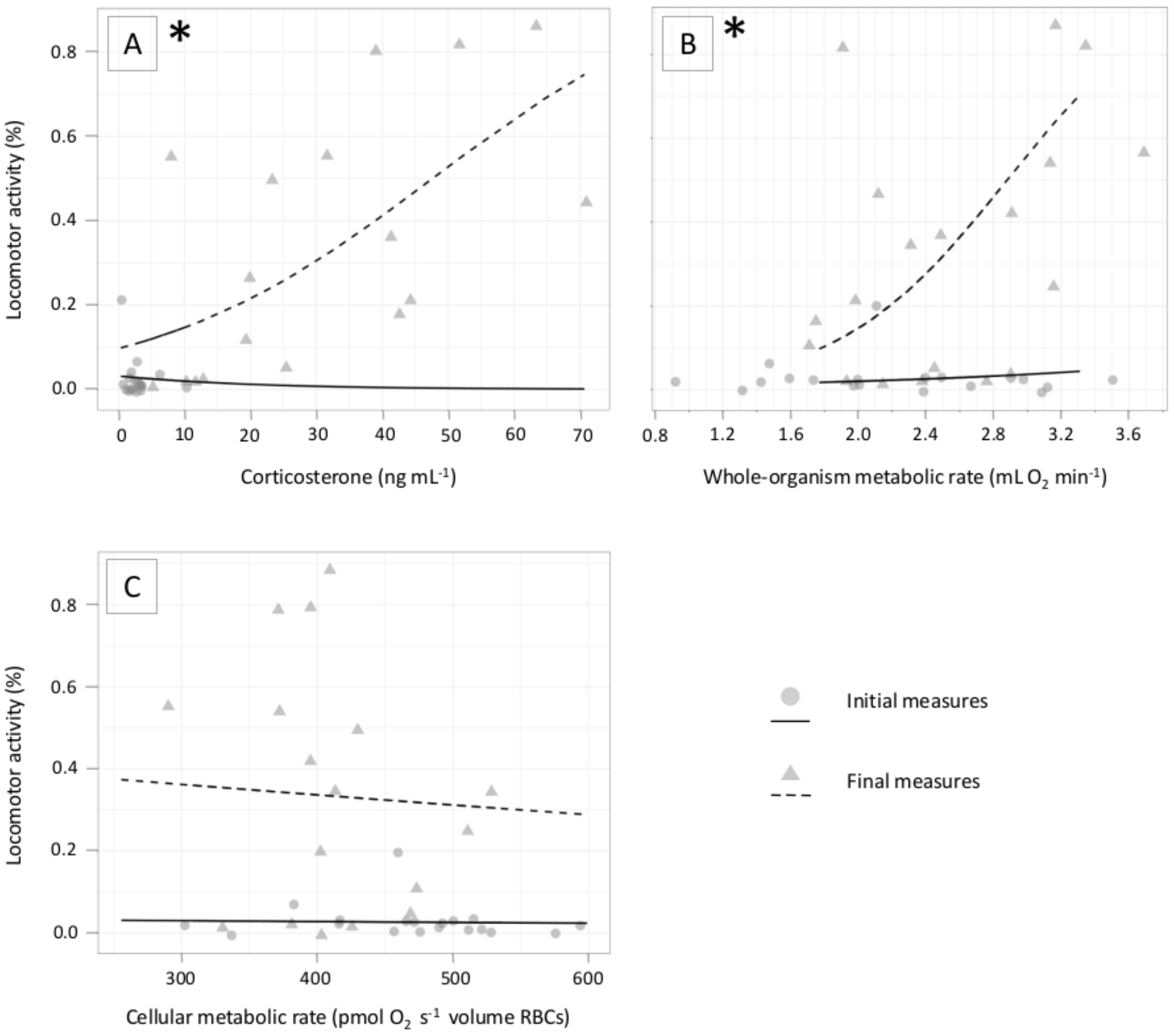

Supplementary Figure 3. Individual variation in locomotor activity during the 2.5 h of respirometry. Activity is expressed as the number of seconds for each minute-interval during which an individual displayed activity (i.e. a value of 60 means that during that minute the individual was always active). Colored lines represent each one individual of the study population. Note that all birds rested during the first ~ 30 min of respirometry. Note also the great variability in activity patterns among individuals during the last 1.5 h.

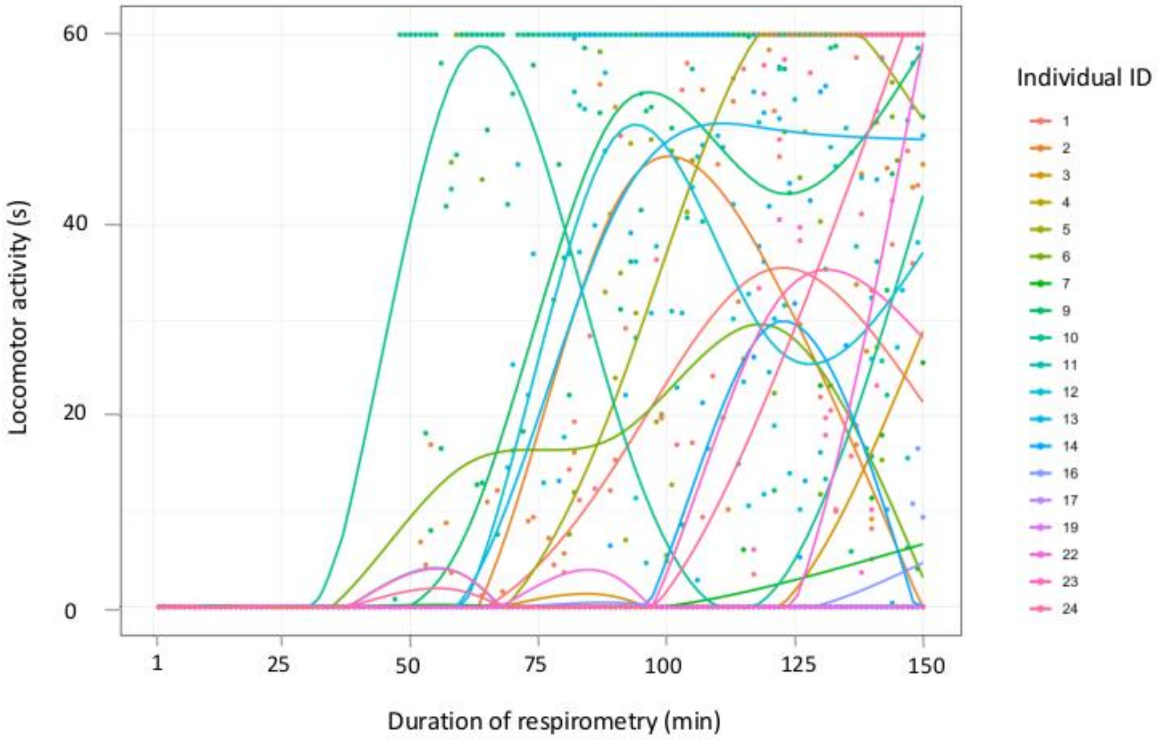

Supplementary Figure 4. Positive relationship between individual responses in whole-organism metabolic rate and corticosterone concentration.

Responses in both traits are calculated as the change from initial to final measure, i.e. the difference between final and initial absolute values. Blue horizontal line indicates no change in woMR. Above this line woMR increased (also indicated by the red arrow) while below it, woMR decreased (green arrow). We can distinguish between *"high-cort"* birds (when the corticosterone increase was around or above stress-induced ranges reported for this species, i.e. ≥ ~20 ng mL^{-1}; Baugh et al., 2013; see Discussion) and *"low-cort"* birds (when corticosterone increased only slightly or not at all, i.e. stayed < ~20 ng mL^{-1}). High-cort individuals showed an increase in woMR, while low-cort individuals showed a decrease in woMR.

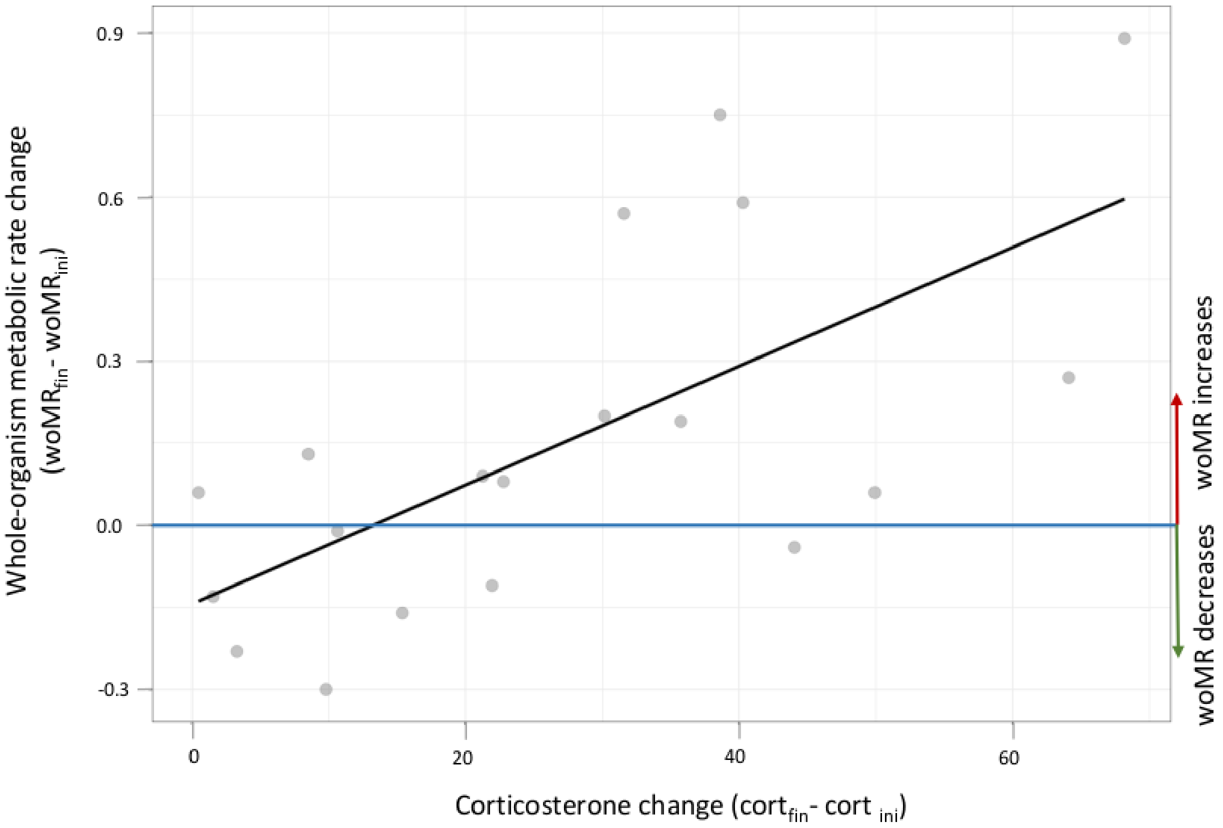

Supplementary Table 1. LMM models to analyze the influence of internal (body mass), external (ambient temperature, Ta) and experimental predictors (measurement, sampling latency to collect blood or metabolic chamber number) on corticosterone concentrations (**A**), whole-organism metabolic rate (**B**), cellular metabolic rate (**C**) and locomotor activity (**D**). Additional random factors were originally included but excluded from the final models when they did not significantly explain variation. Specifically: assay plate and identity of the person collecting blood sample were removed from model A); Oroboros chamber number where RBC O_2 consumption was measured was removed from model C); and video camera number was removed from model D). Statistically meaningful effects are reported in bold. Fixed effects, random effects and residual variance are presented together with their 95% CrI (in brackets). Within each model, continuous predictors were mean centered and their variance standardized to allow among-effect comparisons.

	Corticosterone	*Whole-organism metabolic rate*	*Cellular metabolic rate*	*Locomotor activity*
	A)	B)	C)	D)
Fixed factors	β (95% CrI)	β (95% CrI)	β (95% CrI)	β (95% CrI)
Intercept	0.39 (0.22; 0.55)	**2.47** (2.19; 2.75)	**458.80** (428.67; 488.72)	**-2.15** (-3.23; -2.12)
Mass	-0.04 (-0.17; 0.09)	-0.06 (-0.19; 0.06)	**31.17** (7.58; 55.61)	**-0.33** (-0.79; -0.11)
T_a	-0.06 (-0.18; 0.06)	**-0.24** (-0.35; -0.13)	11.84 (-9.44; 34.86)	**-0.50** (-0.74; -0.35)
Measurement (fin)	**0.92** (0.68; 1.17)	0.03 (-0.14; 0.22)	**-48.55** (-95.47; -3.00)	**1.34** (0.99; 2.22)
Sampling latency	**0.11** (-0.02; 0.23) *	-	-	-
Random factors	σ^2 (95% CrI)	σ^2 (95% CrI)	σ^2 (95% CrI)	σ^2 (95% CrI)
Chamber	-	0.05 (0.00; 0.22)	-	-
Individual ID	0.00 (0.00; 0.00)	0.02 (0.01; 0.04)	0.00 (0.00; 0.00)	0.05 (0.00; 7.07)
Residual	0.12 (0.08; 0.20)	0.07 (0.04; 0.11)	4568.26 (2977.01; 7489.36)	2.59 (0.51; 8.56)

* *Statistically meaningful effect, whose posterior probability is ≥ 95%, despite zero being included in the credible interval*

Supplementary Table 2. Results from LMMs exploring the relationship between corticosterone concentrations and whole-organism metabolic rate (**A**) and cellular metabolic rate (**B**), respectively. Each model contains additional factors shown in earlier analyses to influence the response variable (Supplementary Table 1). Statistically meaningful effects are reported in bold. Fixed effects, random effects and residual variance are presented together with their 95% CrI (in brackets). Continuous predictors were mean centered and their variance standardized to allow for a comparison among effects. Note that woMR is positively affected by final corticosterone concentrations (after respirometry), while cMR is not.

	Whole-organism metabolic rate	*Cellular metabolic rate*
	A)	B)
Fixed factors	β (95% CrI)	β (95% CrI)
Intercept	**2.41** (2.12; 2.71)	**476.34** (420.64; 531.35)
Log_{10}(cort)	-0.05 (-0.25; 0.14)	12.66 (-42.11; 68.19)
Measurement (fin)	**-0.23** (-0.47; 0.00)	**-86.72** (-172.80; -3.40)
Log_{10}(cort) x Measurement (fin)	**0.50** (0.22; 0.79)	-0.36 (-88.12; 88.54)
Mass	0.00 (-0.11; 0.12)	**27.33** (3.29; 51.26)
T_a	**-0.17** (-0.27; -0.07)	17.27 (-6.70; 40.55)
Random factors	σ^2 (95% CrI)	σ^2 (95% CrI)
Chamber	0.04 (0.00; 0.18)	-
Individual ID	0.02 (0.01; 0.03)	0.00 (0.00; 0.00)
Residual	0.04 (0.02; 0.06)	4007.64 (2525.96; 6812.65)

Supplementary Table 3. Results from GLMMs (beta family) to analyze the relationship between locomotor activity displayed during respirometry and corticosterone concentrations (**A**), whole-organism metabolic rate (**B**) or cellular metabolic rate (**C**), depending on measurement (initial or final). Fixed effects, random effects and residual variances are presented together with their 95% CrI (in brackets). Continuous predictors were mean-centered and their variances standardized to facilitate comparisons. CrI that do not overlap zero indicate statistically meaningful effects and are highlighted in bold. All models include ambient temperature and body mass as additional predictors (Supplementary Table 1).

	Locomotor activity	*Locomotor activity*	*Locomotor activity*
	A)	B)	C)
Fixed factors	β (95% CrI)	β (95% CrI)	β (95% CrI)
Intercept	**-3.91** (-4.14; -1.87)	**-2.53** (-3.14; -2.14)	**-2.78** (-2.97; -1.70)
Mass	0.08 (-0.41; 0.22)	-0.15 (-0.40; 0.03)	-0.20 (-0.57; 0.15)
T_a	0.12 (-0.38; 0.24)	0.18 (-0.31; 0.33)	**-0.43** (-0.83; -0.05)
Measurement (fin)	-0.60 (-0.77; 1.28)	**1.75** (1.01; 2.11)	**1.60** (0.85; 2.30)
Log_{10}(cort)	-0.82 (-0.99; 0.71)	-	-
Log_{10}(cort) x measurement (fin)	**1.80** (0.83; 3.15)	-	-
woMR	-	0.43 (-0.35; 0.71)	-
woMR x measurement (fin)	-	**0.59** (0.14; 1.26)	-
cMR	-	-	0.05 (-0.25; 0.78)
cMR x measurement (fin)	-	-	-0.76 (-0.92; 0.44)
Random factors	σ^2 (95% CrI)	σ^2 (95% CrI)	σ^2 (95% CrI)
Individual ID	0.10 (0.00; 7.92)	1.38 (0.00; 7.10)	4.27 (0.00; 14.20)
Residual	0.85 (0.00; 6.63)	0.44 (0.00; 4.14)	0.03 (0.00; 5.44)

Supplementary Table 4. Results from LM investigating the corticosterone-dependent association between woMR and cMR, but using trait changes instead of absolute values. Each trait is expressed as the difference between its final and initial value. Ambient temperature and change in body mass were used as covariates. Fixed effects and residual variance are presented together with their 95% CrI (in brackets). Continuous predictors were mean-centered and their variances standardized to facilitate comparisons. CrI that do not overlap zero indicate statistically meaningful effects and are highlighted in bold.

	Whole-organism metabolic rate change ($woMR_{fin}$-$woMR_{ini}$)
Fixed factors	β (95% CrI)
Intercept	**0.19** (0.09; 0.29)
cMR_{change}	-0.01 (-0.12; 0.12)
$cort_{change}$	**0.23** (0.13; 0.34)
cMR_{change} x $cort_{change}$	**-0.13** (-0.28; 0.01) *
$Mass_{change}$	-0.03 (-0.13; 0.07)
T_a	0.04 (-0.05; 0.14)
Random factors	$σ^2$ (95% CrI)
Residual	0.06 (0.04; 0.11)

** Statistically meaningful effect, whose posterior probability is ≥ 95%, despite zero being included in the credible interval*

Supplementary Text 1. Corticosterone assays

Plasma corticosterone concentrations were measured in duplicate with an enzyme-linked immunosorbent assay (ELISA) kit (Arbor Assays, Catalog No. K014-H5), following a double diethyl ether extraction of 7µL to 14 µL of plasma. Samples were re-dissolved in 120-150 µL assay buffer and reconstituted overnight at 4°C. Two negative controls containing only water were included in each assay step of the extraction protocol to exclude contamination. Values for all negative controls were below the detection limit. Two positive controls containing stripped chicken plasma spiked with a known amount of corticosterone were also included in each assay step, serving as quality control samples. Our low quality control (containing on average 5 ng mL^{-1}) was measured at a 1:30 dilution factor, which corresponds to a binding around 75%, while our high quality control (containing on average 6.5 ng mL^{-1}) was measured at a 1:18.8 dilution factor, which corresponds to a binding around 46%. These samples were used to calculate inter-assay and intra-assay variance (using the coefficient of variance; see main text).

Supplementary Text 2. Whole-organism O_2 consumption calculations

At the end of the experiment, we ran a customized macro (built in Expedata, Sable Systems, Las Vegas, NV, USA) on each data file to apply the necessary corrections and to calculate the rate of O_2 consumption. Specifically, raw data were lag- and response-corrected and excurrent O_2 and CO_2 partial pressures were mathematically corrected for the dilutive effect of water vapor pressure (which in our system is scrubbed upstream but not downstream of the chamber) by applying equation 8.7 in Lighton, 2008:

$$F'_eO_2 = F_eO_2 \times BP / (BP - WVP)$$

$$F'_eCO_2 = F_eCO_2 \times BP / (BP - WVP)$$

Where F_eO_2 and F_eCO_2 are the original O_2 and CO_2 values in the excurrent air, BP is the barometric pressure and WVP is the water vapour pressure, in the same unit (kPa). Finally, the O_2 consumption rate VO_2 was calculated by applying equation 10.5 in Lighton, 2008 for push systems, as follows:

$$VO_2 = FR \times ((F_iO_2 - F'_eO_2) - F'_eO_2 \times (F'_eCO_2))/ (1-F'_eO_2)$$

where FR is the flow rate of dry, CO_2-free air pushed into the metabolic chamber (in mL min^{-1}), F_iO_2 is the partial pressure of O_2 in the incurrent air, F'_eO_2 is the partial pressure of O_2 in the excurrent air after correcting for the dilutive effect of water vapour pressure and F'_eCO_2 is the partial pressure of CO_2 in the excurrent air after applying water vapour pressure dilution correction.

Ultimately, the macro averaged VO_2 values over 1-minute periods allowing us to select the first and last 10 minutes of each recording to extract the mean initial woMR and the mean final woMR for each bird ($woMR_{ini}$ and $woMR_{fin}$, respectively).

Finally, we calibrated the O_2 analyzer every day before the onset of the experiment. Given the fuel cell sensor of the analyzer, the daily O_2 calibration focused on setting the actual O_2 partial pressure of the dry, CO_2-free air in the room to the maximally possible value, i.e., 20.95%. By contrast, H_2O and CO_2 analyzers were calibrated only once, at the beginning of the 8-day experiment, because they are usually very stable. The maximum value for CO_2 was set to 0.977% by using a natural gas and zeroed using nitrogen, whilst H_2O was set to its maximum value by using the dilution observed in O_2 when changing from chemically scrubbed dry CO_2-free air to outdoor unscrubbed air. We did so by applying the following equation:

$$WVP = BP\ (F'_iO_2 - F_iO_2)/F'_iO_2$$

Where F'_iO_2 is the O_2 partial pressure in the chemically scrubbed air-stream (at 20.95% of O_2) and F_iO_2 is the O_2 partial pressure in the unscrubbed air.

Supplementary Text 3. Locomotor activity behavior during respirometry: quantification, analysis and results.

a. *Quantification of activity*

We video-recorded the behavior of each bird using mini infrared video cameras (Handykam, Sony HD, HK100441w, Redruth, United Kingdom) placed inside the environmental cabinet and facing the transparent lid of the metabolic chamber. Video analysis was carried out by KM using Salomon Coder (version beta 16.05.16, developed by Andras Peter) and by employing a focal-animal sampling protocol (Altmann, 1974). We categorized the bird's behavioral state throughout the video in a mutually exclusive manner as either drowsiness, sleep, wakefulness or locomotor activity. Here we only analyzed data on the locomotor activity state ("activity") as a behavioral measure of stress. We coded as activity any instance when the bird moved repeatedly and quickly from one side of the perch to the other, left the perch to hop on the bottom of the chamber, or made stereotyped circular movements around the perch while clinging to it. Active birds often engaged for extended periods of time in preening and pecking of exposed surfaces or their leg bands. Thus, $activity_{ini}$ and $activity_{fin}$ (Figure 1) represent the percent of time spent displaying active behaviors in the first and last hour of respirometry, respectively.

b. *Statistical analysis of activity*

To test if the extent of locomotor activity shown inside the metabolic chamber was related to corticosterone concentrations and metabolic traits of individuals, we first used zero-inflated generalized linear mixed-effects models (ZIGLMMs) with a beta family and logit link. However, due to convergence problems and because information resulting from the zero-inflation part of the model was never meaningful (data not shown), we decided to resort to linear mixed-effects models (GLMMs) without modeling the excess of zeros separately. Activity was fit as the response variable and individual ID as a

random factor to account for individuals being measured twice ($activity_{ini}$ and $activity_{fin}$). Both ZIGLMMs and GLMMs were fit using the “glmmTMB” package (Brooks et al., 2017). To meet the criteria of beta distributions, activity was converted and rescaled from percent of time to range from 0 to 1.

We decided to model locomotor activity separately for the three traits below because explanatory variables varied between initial and final measures, which resulted in chain convergence problems when attempting to fit a full model that included all three predictors, each in interaction with measurement. We thus built three separate GLMMs (Supplementary Table 3) to model activity, first, as a function of corticosterone concentrations depending on measurement (“cort x measurement”), second, as a function of whole-organism metabolic rate depending on measurement (“woMR x measurement”) and, third, as a function of cellular metabolic rate depending on measurement (“cMR x measurement”). Moreover, since ambient temperature and body mass seemed to influence the degree of activity (Supplementary Table 1), we included these two factors in each of the above models.

Simulated values from the joint posterior distribution of model parameters were obtained with Hamiltonian Monte Carlo sampling, with 4 chains run for 8.000 iterations each, with a burn-in of 4.000 iterations, using the “rstan” and “tmbstan” packages (Monnahan and Kristensen, 2018). 95% Bayesian credible intervals (CrI) were extracted around the mode in all GLMMs. Convergence diagnostics in GLMMS were instead performed by calculating the ‘’Rhat’’ statistics (Brooks & Gelman, 1998) and the ratio between the effective size of independent posterior samples over the total number of iterations, which confirmed that posterior distributions of the parameters of interest in all our GLMMs were reliably estimated.

c. *Results on activity*

Most birds showed no or very little activity during the first 30 min of respirometry, after which a large among-individual variability in locomotor activity became apparent, with some individuals displaying high rates of activity and others only little (Supplementary Figure 4). We found a meaningful positive effect of the interaction term ‘*’woMR x measurement*’’ which indicates that birds that displayed a high rate of activity in the second part of the respirometry also showed a higher woMR (*β= 0.59; CrI= (0.14; 1.26);* Supplementary Table 3B, Supplementary Figure 3B). Moreover, we found a meaningful positive effect of the interaction term “*cort x measurement*’’ indicating that highly active individuals in the second part of the experiment increased corticosterone concentrations to a great extent (*β= 1.80; CrI= (0.83; 3.15);* Supplementary Table 3A, Supplementary Figure 3A). This finding parallels the relationship between woMR and corticosterone (Supplementary Table 2A and Supplementary Figure 5), suggesting that corticosterone and locomotor activity may both represent stress responses to respirometry. The absence of a relationship between cMR and activity levels (Supplementary Table 3C,

Supplementary Figure 3C) also parallels the lack of a relationship between cMR and corticosterone, and suggests that cMR was not associated with this behavioral correlate of stress.

Supplementary Text 4. Further information on statistical analyses

a. Model diagnostics

LMM model fit was confirmed by visual inspection of model residuals. Moreover, we are aware that including many factors (n=8 in our main LMM; Table 1) in models run on small datasets (in our case n=21, obs=42) may induce overfitting. Overfitting can be diagnosed by checking the degree of uncertainty in parameter estimates, i.e. the 95% CrI (Korner-Nievergelt et al., 2015). In our case, the 95% CrI around the parameters of interest remained entirely within the range of the raw data, thus supporting our confidence in the model output (Figure 3). Additionally, overfit models tend to describe random noise around data points, leaving no or little random error between fitted lines and the observed values. By visually inspecting plots of residuals versus fitted values we made sure that this was not the case for our data.

b. Variables excluded from the full LMM

Besides the full model structure presented in the main text (Table 1 and ‘’Material and Methods’’ section) we also initially included measurement in a three-way interaction with cMR and corticosterone ("*cMR x cort x measurement*") to test whether the cort-mediated woMR-cMR association differed between initial and final measures. Nevertheless, as the interaction term resulted in a non-meaningful effect (*β=-0.17; CrI= (-0.66, 0.32))* with a posterior probability far lower than 95% and the BIC resulted in a higher value (64.27 *versus* 57.23), we did not retain the three-way interaction term in the final model. Likewise, we initially also included "sex" to account for any sex difference, but this term was not influential either and therefore was not retained in the final model.

Reference list

Altmann, J. (1974). Observational Study of Behavior: Sampling Methods. *Behaviour* 49, 227–267.

Baugh, A. T., van Oers, K., Naguib, M., and Hau, M. (2013). Initial reactivity and magnitude of the acute stress response associated with personality in wild great tits (Parus major). *General and Comparative Endocrinology* 189, 96–104. doi:10.1016/j.ygcen.2013.04.030.

Brooks, M. E., Kristensen, K., van Benthem, K. J., Magnusson, A., Berg, C. W., Nielsen, A., et al. (2017). glmmTMB balances speed and flexibility among packages for zero-inflated generalized linear mixed modeling. *The R journal* 9, 378–400.

Brooks, S. P., and Gelman, A. (1998). General methods for monitoring convergence of iterative simulations. *Journal of computational and graphical statistics* 7, 434–455.

Korner-Nievergelt, F., Roth, T., Von Felten, S., Guélat, J., Almasi, B., and Korner-Nievergelt, P. (2015). *Bayesian data analysis in ecology using linear models with R, BUGS, and Stan*. Amsterdam: Acad. Press, an imprint of Elsevier.

Lighton, J. R. B. (2008). *Measuring Metabolic Rates*. Oxford University Press doi:10.1093/acprof:oso/9780195310610.001.0001.

Monnahan, C. C., and Kristensen, K. (2018). No-U-turn sampling for fast Bayesian inference in ADMB and TMB: Introducing the adnuts and tmbstan R packages. *PLoS ONE* 13, e0197954. doi:10.1371/journal.pone.0197954.
